# Antileukemic Effect of Palladium Nanoparticles Mediated by White Tea (*Camellia sinensis*) Extract *In Vitro* and in WEHI-3B-Induced Leukemia *In Vivo*

**DOI:** 10.1155/2020/8764096

**Published:** 2020-08-19

**Authors:** Hemn Othman, Heshu Rahman, Syam Mohan, Sadat Aziz, Hardi Marif, Dianne Ford, Nozlena Abdulsamad, Kawa Amin, Rasedee Abdullah

**Affiliations:** ^1^Faculty of Veterinary Medicine, Universiti Putra Malaysia, UPM Serdang, Selangor, Malaysia; ^2^College of Pharmacy, University of Sulaimani, Sulaymaniyah, Kurdistan Region, Iraq; ^3^College of Medicine, University of Sulaimani, Sulaymaniyah, Kurdistan Region, Iraq; ^4^College of Health Sciences, Komar University of Science and Technology, Chaq-Chaq Qularaisee, Sarchinar District, Sulaymaniyah, Kurdistan Region, Iraq; ^5^Substance Abuse and Toxicology Research Center, Jazan University, Jazan, Saudi Arabia; ^6^College of Veterinary Medicine, University of Sulaimani, Sulaymaniyah, Kurdistan Region, Iraq; ^7^Faculty of Health & Life Sciences, Northumbria University, Newcastle upon Tyne, UK; ^8^Integrative Medicine Cluster, Institut Perubatan dan Pergigian Termaju (IPPT), Sains@BERTAM, Universiti Sains Malaysia, Kepala Batas, Pulau Pinang, Malaysia; ^9^Department of Medical Science, Respiratory, Allergy and Sleep Research, Uppsala University, Uppsala, Sweden

## Abstract

This study investigated the *in vivo* antileukemic activity of palladium nanoparticles (Pd@W.tea-NPs) mediated by white tea extract in a murine model. The cell viability effect of Pd@W.tea-NPs, “blank” Pd nanoparticles, and white tea extract alone was determined in murine leukemia WEHI-3B cells and normal mouse fibroblasts (3T3 cells). Apoptotic and cell cycle arrest effects of Pd@W.tea-NPs in WEHI-3B cells were evaluated. The effects of Pd@W.tea-NPs administered orally to leukemic mice at 50 and 100 mg/kg daily over 28 days were evaluated. Pd@W.tea-NPs reduced the viability of WHEI-3B cells with IC_50_ 7.55 *μ*g/ml at 72 h. Blank Pd nanoparticles and white tea extract alone had smaller effects on WHEI-3B viability and on normal fibroblasts. Pd@W.tea-NPs increased the proportion of Annexin V-positive WHEI-3B cells and induced G2/M cell cycle arrest. Leukemic cells in the spleen were reduced by Pd@W.tea-NPs with an increase in Bax/Bcl-2 and cytochrome-C protein and mRNA levels indicating the activation of the mitochondrial apoptotic pathway. These effects replicated the effects of ATRA and were not observed using blank Pd nanoparticles. Pd@W.tea-NPs afford therapeutic efficacy against leukemia likely to pivot on activation of the mitochondrial pathway of apoptotic signaling and hence appear attractive potential candidates for development as a novel anticancer agent.

## 1. Introduction

A substantial proportion of 10 million new cancers reported globally each year, and of those, 6 million annual cancer deaths are due to leukemia [1]. It is estimated by the National Cancer Institute that leukemia will account for 3.5% (over 60,000) of new cancers in the US population in 2019 and for 3.8% of all cancer deaths [2]. Conventional therapies such as imatinib mesylate (Glivec), all-trans retinoic acid (ATRA), arsenic trioxide (ATO), and doxorubicin (DOX) can be highly effective, especially in newly diagnosed acute leukemias. However, the chances of relapse, fatal side effects, and development of multidrug resistance are high [3]. Thus, less toxic and more target-specific therapies are desirable. In this regard, dependable, environmentally benign processes for the synthesis of nanoscale materials that simultaneously confer additional beneficial properties through the incorporation of natural molecules, such as antioxidants, from the reaction mixture is a promising development in nanomedicine for the treatment of cancer, including leukemia [4–6]. The use of bioresources, including plant products, in the synthesis of metal nanoparticles shows promise in this regard, as well as in providing a viable and cost-effective approach with minimal environmental impact for a wide range of other potential applications, including in optics and biomedicine. The activities of natural chemical reductants in plants with ethnobotanical relevance that also have reported antimicrobial, anticancer, antioxidant, and/or anti-inflammatory activities have been harnessed to synthesize palladium nanoparticles (Pd-NPs). These include cinnamon (*Cinnamomum camphora*) leaf [7], black tea (*C. sinensis*) leaf [8], fenugreek (*Trigonella foenum-graecum*) [9], gum ghatti (*Anogeissus latifolia*) [10], *Chlorella vulgaris* [11], white tea (*C. sinensis*) [12, 13] citrus fruit (*Cymbopogon citratus*) [14], and barberry fruit [15].

Tea, which belongs to the family Theaceae, is mainly planted in China, India, and, to a lesser extent, in tropical regions. Time of harvesting and processing is generally species-specific and confers different qualities and properties. White tea (*Camellia sinensis*), which is harvested before the leaves open fully, when the young buds are still covered by fine white hairs [12], has been used as a traditional treatment for various diseases including diabetes, inflammation, obesity, aging, osteoporosis, and cancer [16]. It is well-known for its high content of polyphenols, particularly catechin [13, 16, 17]. These chemicals are known to have wide spectrum of biological effects such as antioxidant, anticancerous, antiviral, and antifungal effects [18, 19].

Previously, we reported the first synthesis of a Pd-NP composite using white tea extract. The *in vitro* antioxidant, antibacterial, and antiproliferative activities toward the human leukemia (MOLT-4) cell line were enhanced compared with normal human fibroblasts [7]. To our knowledge, the effect of white tea extract-derived Pd-NPs (Pd@W.tea-NPs) on leukemic cells *in vivo* has not been reported. Thus, we investigated the antileukemic effects of Pd@W.tea-NPs *in vivo* using a murine model of acute myelocytic leukemia. We also confirmed that our previous finding that these nanoparticles have activity *in vitro* (in human leukemia cells) that is enhanced compared with normal human fibroblasts applies also in murine leukemia cells (WEHI-3B) compared with normal murine fibroblasts.

## 2. Materials and Methods

### 2.1. Preparation and Characterization of Palladium Nanoparticles

Palladium nanoparticles were prepared and characterized as described previously [7]. In brief, 50 ml of freshly prepared clear extract of white tea was dispersed in a flask containing 50 ml of 1 mM PdCl_2_ solution at 40°C with constant stirring. Over ∼30 min, the colour of the reaction contents gradually changed from transparent yellow to dark brown, indicating successful formation of Pd-NPs. The reaction was allowed to continue for 2 h. The product was then concentrated by centrifugation at 6000 rpm for 10 min. Finally, the sample was oven-dried (60°C), until it had the appearance of a fine powder, and then stored in a clean glass container. “Blank” palladium nanoparticles were prepared in the absence of white tea extract. Pd@W.tea-NPs were characterized using UV-visible spectrophotometry (Lambda 25-Perkin-Elmer, Waltham, MA, USA), Fourier-transform infrared (FTIR) spectrometry (Perkin-Elmer 1725X), X-ray diffraction (XRD-6000; Shimadzu), transmission electron microscopy (Hitachi H-7650, Tokyo, Japan), and scanning electron microscopy (Philips XL-30, Germany).

### 2.2. Measurement of Anticancer Activity *In Vitro*

#### 2.2.1. Cell Lines and Culture Conditions

Murine myelocytic leukemia (WEHI-3B) and murine fibroblast (3T3) cell lines were purchased from the American Type Culture Collection (ATCC) (Manassas, VA, USA). Cells were maintained in complete RPMI-1640 medium supplemented with 10% FBS and 1% antibiotic-antimycotic in 75 cm^2^ culture flasks (TPP, Trasadingen, Switzerland) at 37°C and 5% CO_2_ in a humidified incubator (Binder, Tuttlingen, Germany).

#### 2.2.2. Cytotoxicity Assay

The cytotoxic effect of Pd@W.tea-NPs, blank Pd-NPs, and white tea extract alone on WEHI-3B and 3T3 cells was measured using MTT assay. Cells were seeded at a density of 1 × 10^4^ cells per well in 1 ml RPMI-1640 or DMEM medium, respectively, in 96-well plates and exposed to test agents 24 h after seeding. Then, they were kept for 24, 48, or 72 h in a CO_2_ incubator. MTT (5 mg/ml in PBS) was then added, and cells were incubated for further 3 h at 37°C. DMSO was added to reduce tetrazole to formazan, and concentration was measured at 570 nm (Bio-Rad, Model 680). IC_50_ was calculated by comparison with unexposed cells.

#### 2.2.3. Detection of Apoptotic Cells Using FITC-Annexin V and Propidium Iodide

WEHI-3B cells (∼1 × 10^6^) were seeded in a 25 cm^2^ flask and then treated with Pd@W.tea-NPs at IC_50_ for 24, 48, or 72 h. Cells grown in culture medium without Pd@W.tea-NPs were used as controls at each time point. Cells were then stained with Annexin V and propidium iodide (PI) using the apoptosis detection kit (BD Biosciences, San Jose, CA, USA) according to the manufacturer's instructions. Annexin V-FITC and PI binding were analyzed immediately by flow cytometry (FACS Calibur, BD Biosciences, San Jose, CA, USA). Cell Quest software was used for quadrant analysis.

#### 2.2.4. Cell Cycle Assay

Cell seeding and treatment for measurement of the cell cycle was as described for measurement of apoptosis. Cells were harvested, fixed, and permeabilized with 70% ethanol at −20°C for at least 72 h, washed and resuspended in 0.5 ml PBS containing propidium iodide (1 mg/ml) and RNases (0.5 mg/ml), and then incubated for at least 30 min at 4°C in the dark. Finally, the DNA content was quantified by flow cytometry (FACS Calibur (BD Biosciences, San Jose, CA, USA) using BD FACS Diva software for data analysis as reported previously [20].

### 2.3. Measurement of Anticancer Activity *In Vivo*

#### 2.3.1. Animals

Thirty-six male BALB/c mice, 6–8 weeks old, with an average body weight of 20–25 g, were supplied by the Animal House, Faculty of Veterinary Medicine, Universiti Putra Malaysia (UPM). The animals were kept in 12 h light and dark cycles at a controlled temperature of 25 ± 2°C and fed regularly with mouse chow. Mice were housed for a week under these conditions to allow acclimatization before treatment. The study was approved by the Animal Care and Use Committee (ACUC), Faculty of Veterinary Medicine, Universiti Putra Malaysia (ref.: UPM/IACUC/AUP-R055/2017).

#### 2.3.2. Animal Grouping, Cancer Induction, and Treatment

Mice were allotted into 6 groups (*n* = 6), namely, I: negative control, II: cancer control, III: treatment 1, IV: treatment 2, V: blank Pd-NP, and VI: positive control. Leukemia allografts were then established by injecting 1 × 10^6^ WEHI-3B cells suspended in 300 *μ*l ice-cold PBS intraperitoneally into the abdomen of each mouse except those in group I. Mice were tested daily for the development of leukemia by the detection of leukemic cells in a tail vein blood smear using Wright stain. Once leukemia was established, several days after the injection of WEHI-3B cells, treatments were administered daily for 4 weeks by oral dosage of 300 *μ*l after 12 h without food using a syringe and ball-tip stainless-steel needle. Group II received saline only; group III received Pd@W.tea-NPs at 50 mg/kg; group IV received Pd@W.tea-NPs at 100 mg/kg; group V received Blank Pd-NPs at 100 mg/kg; and group VI received ATRA (5 mg/kg). Group I received no oral dose. The condition of animals was monitored daily. At the end of the study, animals were anesthetized by intraperitoneal injection of ketamine (83 mg/g) and xylazine (13 mg/g) and then sacrificed. The spleen was collected for macroscopic and microscopic analysis.

#### 2.3.3. Serum Biochemistry

Blood samples were collected by heart puncture. Serum was collected and analyzed immediately using standard diagnostic kits (Roche) in an automatic biochemistry analyser (Hitachi 902, Japan).

#### 2.3.4. Measurement of Spleen

The size of the spleen collected from each animal was measured immediately using a caliper, and then, the spleens were weighed and photographed.

#### 2.3.5. Histopathological Examination of the Spleen

Spleens were washed with normal saline, fixed in 10% formalin, passed through ascending concentrations of ethanol (50, 70, 90, and 100%), embedded in paraffin wax, and then sectioned. Before staining, sections were cleared of wax in 2 changes of xylene for 3 min each, hydrated by passing through descending concentrations of ethanol (2 changes each at 100, then 70%), and rinsed with water for 3 min. Sections were then stained with hematoxylin and eosin (H&E). After staining, sections were dehydrated in ascending concentrations of ethanol (2 changes each for 3 min at 70, then 100%) and then cleared in 2 changes of xylene for 3 min each. Histopathological features, comprising vessel congestion, red blood cell extravasation, hematoma, cell necrosis, nuclear changes, and fibrosis, were scored in 4 sections from each mouse by a blinded observer, and the spleens were graded as 0 (no distinguishable change); 1 (mild change—up to 30%); 2 (moderate change—31–60%); and 3 (severe change—61–100%). Data were analyzed using the Mann–Whitney test (SPSS 16.0), taking *P* < 0.05 as significant change.

#### 2.3.6. Immunohistochemical Examination of the Spleen

The Dako Envision® + Dual Link System-HRP (DAB+) kit (Dako K4965, USA) was used, with a slight modification of the protocol, to detect apoptotic cells in the spleen. Tissue sections, prepared as above, were deparaffinized, rehydrated with ascending concentrations of ethanol (100, 90, and 70%), and washed in distilled water. Wax-enclosed sections were then flooded with dual endogenous enzyme (Dako K4065, USA) as a blocking agent and incubated for 10 min. Sections were then washed with citrate buffer solution (10 mM, pH 6.0) (Sigma, USA), immersed in tris-buffered saline with Tween-20 (TBST) for 3 min, and then incubated with CD3 primary antibody (T-lymphocyte marker) (Abcam ab5690, UK) or CD19 primary antibody (B-lymphocyte marker) (Bioss bs0079R, USA) at 4°C overnight. After washing with TBST, sections were incubated for 45 min with labeled polymer-HRP reagent (Dako K4065, USA) and then washed again with TBST. DAB + substrate-chromogen solution (Dako K4065, USA) was then applied for 3 min. Finally, sections were counterstained with Myer's hematoxylin, mounted in DPX medium, and observed under a light microscope at 40x magnification.

#### 2.3.7. Western Blotting

Total protein was extracted from splenic tissues using RIPA buffer (Sigma-Aldrich, USA) supplemented with protease inhibitor cocktail (Sigma Aldrich Co, LLC, USA). Samples of 20 *μ*g were separated by sodium dodecyl sulfate-polyacrylamide gel electrophoresis (SDS-PAGE) and then transferred to PVDF membranes. Membranes were probed using specific primary antibodies against GAPDH (Abcam ab8245), Bcl-2 (Abcam ab59348), Bax (Abcam ab32503), or cytochrome-C (Abcam ab13575) at 4°C overnight. After washing 3 times (for 5 min each) in TBST, the ALP-conjugated goat anti-rabbit secondary antibody (Bio-Rad ab20571 for anti-Bcl-2 and anti-Bax primaries) or goat anti-mouse secondary antibody (Bio-Rad 170-6464 for anti-GAPDH anti-cytochrome-C primary) was applied, and membranes were then incubated with NBT-BCIP buffer for 15 min in the dark. Protein bands were visualized and captured using a Bio-Rad Gel Doc system and quantified by densitometry using molecular imaging software (ImageJ 148-JDK 6 software).

#### 2.3.8. RT-qPCR

Spleens were placed in RNAlater (Ambion, USA) overnight at 4°C before being minced and then stored in a freezer at −80°C. Tissues were snap-frozen in liquid nitrogen and crushed to a powder before total RNA was extracted using the Qiagen RNAeasy Mini Kit (Qiagen, Germany) and quantified using a nanospectrophotometer (Beckton Coulter, USA). Adequate purity was confirmed based on A260/A280 ratios of 1.8–2.1. Reverse transcription to cDNA was achieved using the Maxima First Strand cDNA synthesis kit (Thermo Scientific, USA). Quantity of Bax, Bcl-2, cytochrome-C, and GAPDH mRNA was measured using SYBR Select Master Mix (Life Technologies, USA) in an Eco Illumina instrument (Illumina, USA) using thermal cycling parameters (optimised for each primer pair) of 95°C for 10 min and then 40 cycles of 95°C for 15 s and 55–60°C for 15–30 s. Quantity of Bax, Bcl-2, or cytochrome-C mRNA was expressed in relation to GAPDH using standard curves for each mRNA and a delta-Ct relative quantification model with PCR efficiency correction using Eco Study software (Illumina, USA). The target cDNA was amplified using the following primers: Bcl-2-forward 5′-CCAGACTCATTCAACCAGACA-3′ and reverse 5′-GATGACTGAGTACCTGAACCG-3′; Bax-forward 5′-TTTGCTACAGGGTTTCAT-3′ and reverse 5′-CTCCATATTGCTGTCCAG-3′; cyt-C forward 5′-GTCTTATGCTTGCCTCCCTT-3′ and reverse 5′-CGTCTGTCTTCGAGTCCGA-3′; and GAPDH forward 5′-CGGGACCTAATGAAACTCCA -3′ and reverse 5′-AATCTCCACTTTGCCACTGC-3′.

### 2.4. Statistical Analysis

All data points determined by western blotting or RT-qPCR were measured in at least triplicate and expressed as mean ± SEM. Statistical significance was evaluated by one-way ANOVA (using Origin 6.1, Northampton, MA) followed by Tukey's B-test, considering *P* < 0.05 as significant.

## 3. Results

### 3.1. Cytotoxic Activity

**Figure 1:**
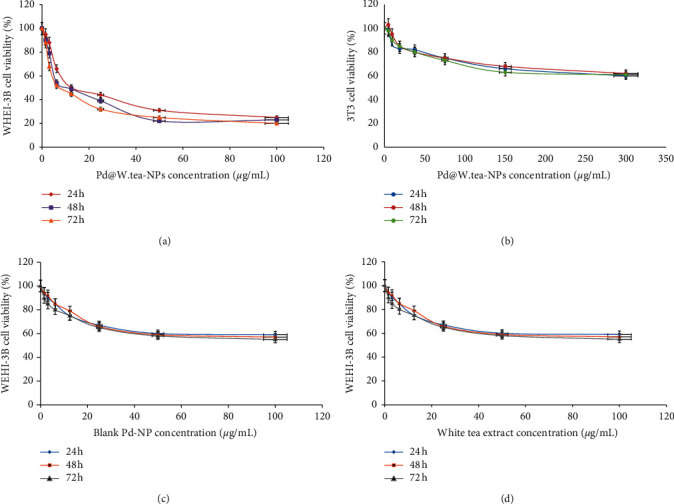
Effects of increasing concentrations of Pd@W.tea-NPs (a, b), blank NPs (c), or white tea extract (d) on the viability of murine myelocytic leukemia cells (WEHI-3B; (a, c, d)) or normal mouse fibroblasts (3T3; b) measured using the MTT assay. The values were expressed as a percentage in relation to the negative control group. Cells were treated for 24, 48, or 72 h as indicated. NPs: nanoparticles.

Pd@W.tea-NPs had a cytotoxic effect on WEHI-3B cells that was dose-dependent and observed as early as 24 h after exposure. Cytotoxicity increased with time up to at least 72 h, at which point cell viability was reduced to 20% (Figure 1(a)). IC_50_ values at 24, 48, and 72 h were 16.77, 10.25, and 7.55 *μ*g/ml, respectively. In contrast, viability of normal mouse fibroblast (3T3) cells was reduced only to 60% by the same maximal exposure (Figure 1(b)). Compared with Pd@W.tea-NPs, both “blank” Pd-NPs and white tea extract had a smaller effect on the viability of WEHI-3B cells, whereas Pd@W.tea-NPs reduced viability to as low as 20% over concentrations increasing to 100 *μ*g/ml, and the same concentration range of the blank NPs or white tea extract achieved only a 50% reduction (Figures 1(c) and 1(d)). Thus, we confirmed that, as observed previously in human MOLT-4 leukemic cells [6], Pd@W.tea-NPs were more effective than white tea extract in reducing the viability of mouse WEHI-3B leukemic cells and also had a differential effect on the leukemic cell line compared with normal fibroblasts. We also demonstrated that Pd@W.tea-NPs had a greater antileukemic action than “blank” NPs in this *in vitro* model.

### 3.2. Apoptosis Analysis

**Figure 2:**
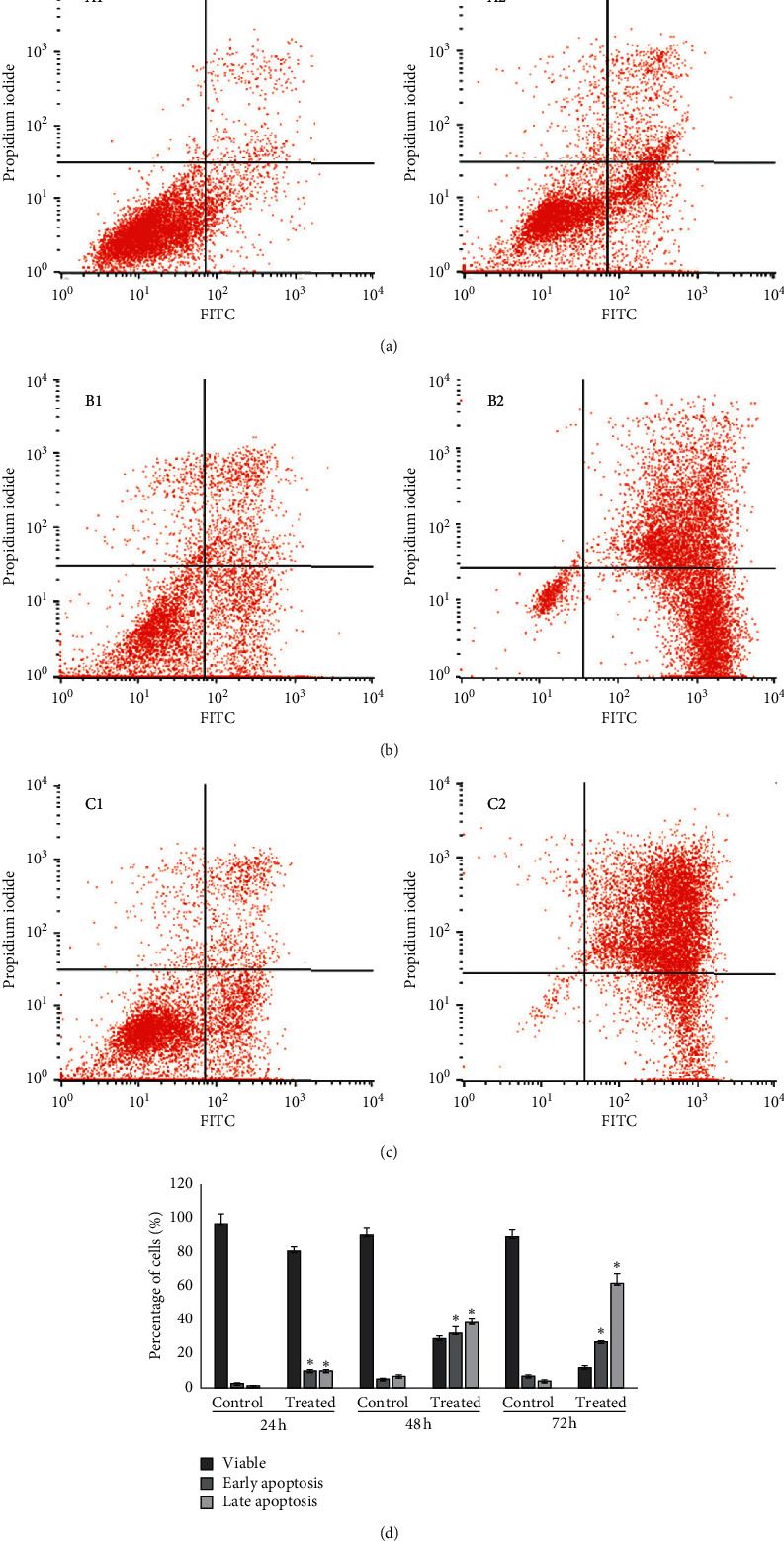
Induction of apoptosis in murine myelocytic leukemia (WEHI-3B) cells by Pd@W.tea-NPs after exposure for (a) 24, (b) 48, or (c) 72 h at IC_50_ (16.77 *μ*g/ml). Apoptosis was detected by staining with Annexin V-FITC followed by flow cytometry. Panels A1, B1, and C1 show untreated (control) cells, while panels A2, B2, and C2 show cells treated with Pd@W.tea-NPs. (d) Quantitative analysis of the flow cytometer data. Values are mean ± SD for three different independent experiments. ^*∗*^*P* < 0.05 by one-way ANOVA followed by Tukey's B-test.

Flow cytometric analysis revealed that treatment of WEHI-3B cells with Pd@W.tea-NPs increased the percentage of Annexin V-FITC-positive cells, indicating in increase in both early and late apoptosis. This effect was dose-dependent, and most marked after longer exposures (Figure 2). At the IC_50_ of 7.55 *μ*g/ml for 72 h exposure, there was an obvious and significant (*P* < 0.05) increase in the percentage of Annexin V-FITC-positive apoptotic WEHI-3B cells compared with untreated control cells (Figure 2).

**Figure 3:**
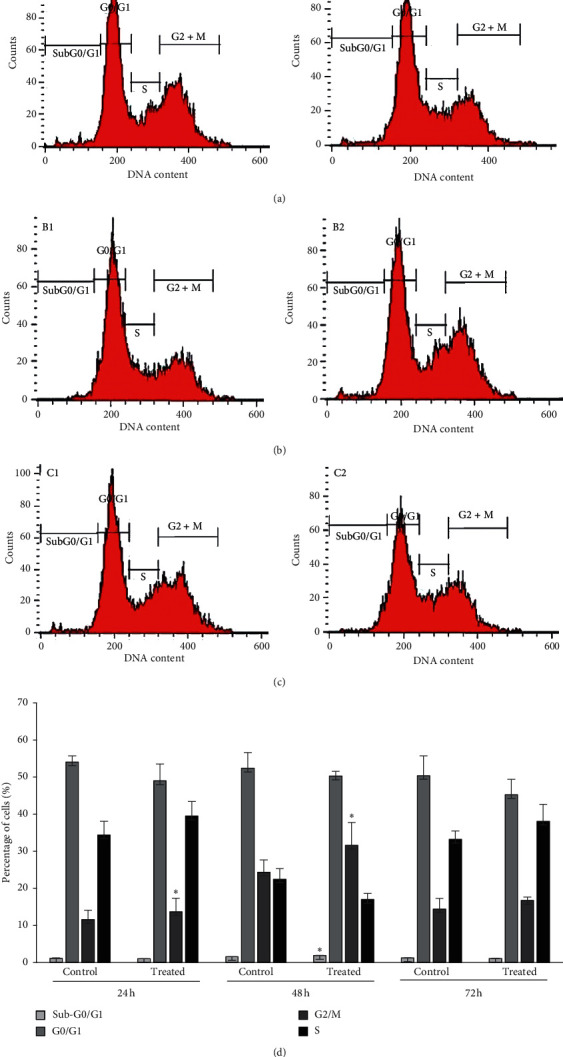
Analysis by flow cytometry of WEHI-3B cells treated with Pd@W.tea-NPs at 7.55 *μ*g/ml (IC_50_ for 72 h exposure) for (a) 24, (b) 48, and (c) 72 h and then stained with propidium iodide. Panels A1, B1, and C1 are untreated control groups. Panels A2, B2, and C2 are cells treated with Pd@W.tea-NPs. (d) Qualitative data of flow cytometer. Values are mean ± SD for three different independent experiments. ^*∗*^*P* < 0.05 by one-way ANOVA followed by Tukey's B-test.

The distribution of WEHI-3B cells across the phases of cell cycle was measured by flow cytometry after treatment with Pd@W.tea-NPs at 72 h IC_50_ of 7.55 *μ*g/ml at 24, 48, and 72 h (Figure 3). The results showed that Pd@W.tea-NPs induced G2/M phase arrest (Figure 3(d)). The number of cells in the G2/M phase was increased significantly (*P* < 0.05) at 24 and 48 h. At 48 h, there was also a significant (*P* < 0.05) increase in the sub-G0/G1 population of cells exposed to Pd@W.tea-NPs.

### 3.3. Biochemical Indicators of Renal and Liver Function

Both serum liver enzymes and renal function parameters were significantly (*P* < 0.05) increased in both the leukemia control and blank NPs groups compared with negative control mice. In contrast, all parameters in the Pd@W.tea-NPs- and ATRA-treated groups were significantly decreased (*P* < 0.05) compared with the induced leukemia control group (Table 1).

ATRA treatment and also treatment with NPs at the higher concentration for some parameters restored these measures to negative control levels, demonstrating that the pathological features the mouse model of leukemia were not simple due to over toxicity.

### 3.4. Morphological Characterization of the Spleen

Treatment of mice in which leukemia was induced by intraperitoneal injection of WEHI-3B (mouse leukemic) cells with Pd@W.tea-NPs reduced the size of the spleen, commensurate with an anticancer effect, and was also observed for ATRA (positive control). Length of the spleen was reduced significantly (*P* < 0.05) after treatment with Pd@W.tea-NPs at both the higher and lower doses tested. In contrast, blank NPs did not reduce spleen length (Table 2). The weight of the spleen in the groups treated with Pd@W.tea-NPs was also reduced significantly (*P* < 0.05) at both the higher and lower doses. In contrast, blank NPs did not reduce spleen weight (Table 2).

### 3.5. Microscopic Characterization of the Spleen

**Figure 4:**
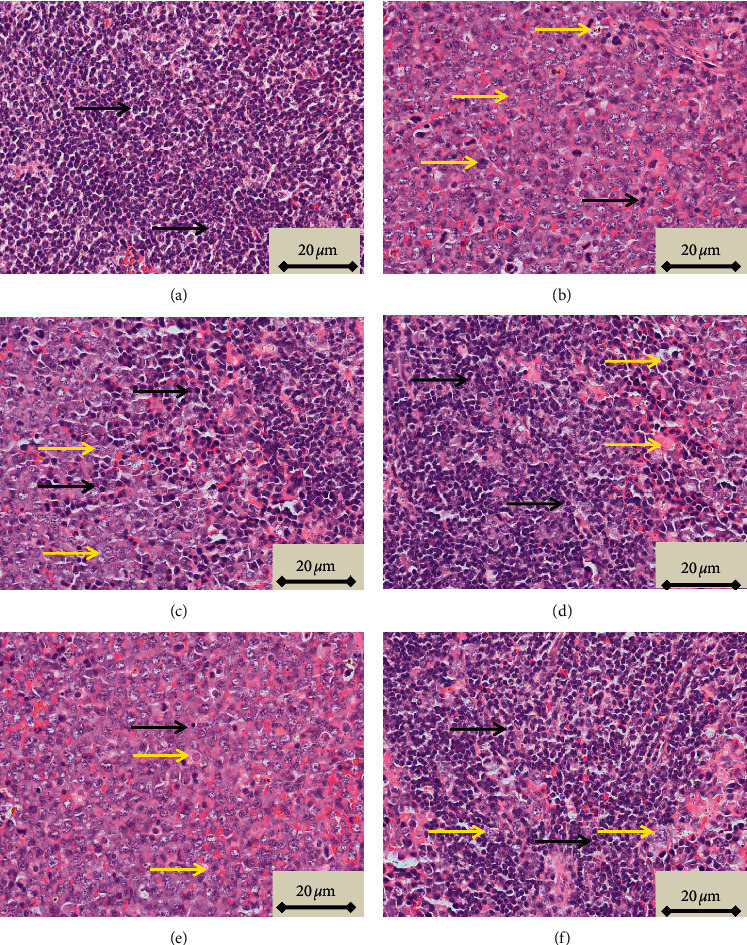
H&E staining (×1000 magnification) of spleen from healthy, control BALB/c mice (a); mice with induced leukemia (b); mice with induced leukemia treated with 50 mg/kg Pd@W.tea-NPs (c); mice with induced leukemia treated with 100 mg/kg Pd@W.tea-NPs (d); mice with induced leukemia treated with 100 mg/kg blank Pd-NPs (e); and mice with induced leukemia treated with ATRA (f). Black arrows indicate examples of cells scored as morphologically normal. Yellow arrows indicate examples of cells scored as neoplastic.

**Figure 5:**
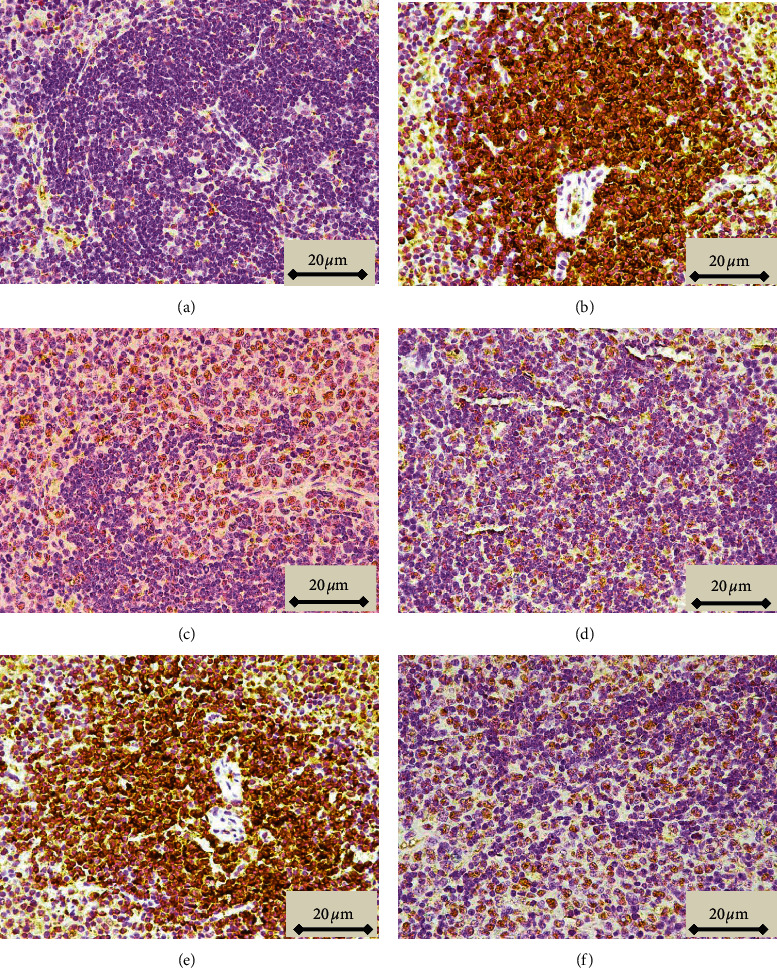
Detection of the CD3 T-cell marker by immunohistochemistry (×1000 magnification) in spleen from healthy, control BALB/c mice (a); mice with induced leukemia (b); mice with induced leukemia treated with 50 mg/kg Pd@W.tea-NPs (c); mice with induced leukemia treated with 100 mg/kg Pd@W.tea-NPs; (d); mice with induced leukemia treated with 100 mg/kg blank Pd-NPs (e); and mice with induced leukemia treated with ATRA (f).

**Figure 6:**
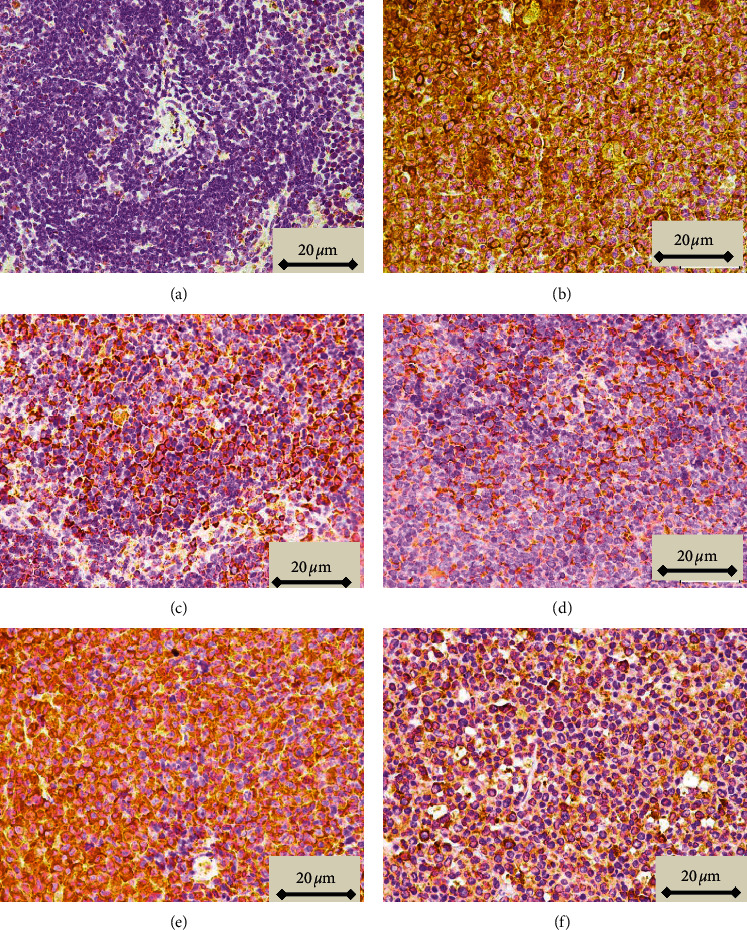
Detection of the CD19 B-cell marker by immunohistochemistry (×1000 magnification) in spleen from healthy, control BALB/c mice (a); mice with induced leukemia (b); mice with induced leukemia treated with 50 mg/kg Pd@W.tea-NPs (c); mice with induced leukemia treated with 100 mg/kg Pd@W.tea-NPs (d); mice with induced leukemia treated with 100 mg/kg blank Pd-NPs (e); and mice with induced leukemia treated with ATRA (f). Cells positive for CD19 are stained brown. Cells negative for CD3 are blue.

Neoplastic cells observed in tissues stained with H&E, characterized by large irregular nuclei with clumped chromatin, clear and light eosinophilic cytoplasm, and irregularity in size and shape, were observed in the red and white pulp in sections of spleen taken from animals in which leukemia was induced and led to the disappearance of sinusoids (Figure 4, compare panels A and B). These histopathological features were reduced in animals treated with both the lower and higher doses of Pd@W.tea-NPs (Figures 4(c) and 4(d)), as well as by ATRA (Figure 5(f)). In contrast, blank NPs did not affect the neoplastic appearance of the tissue (Figure 4(e)). The percentage of cells scored as neoplastic differed significantly (*P* < 0.05) between sections of spleen from healthy control mice and mice with induced leukemia that did not receive NPs and between healthy control mice and mice with induced leukemia treated with blank NPs. The percentage of neoplastic cells also differed significantly (*P* < 0.05) between mice with induced leukemia that did not receive NPs and mice treated with either dose of Pd@W.tea-NPs or with ATRA (Table 3). T and B lymphocytes observed in spleen by immunohistochemical detection of the CD3 T-cell marker [21] and the CD19 B-cell marker18 showed significant differences between treatment groups that were completely consistent with the differences revealed by H&E staining, with CD3-positive cells (T cells) being in the white pulp (Figure 5) and CD19-positive cells (B cells) being in the red pulp (Figure 6).

### 3.6. Determination of Apoptosis Biomarkers

**Figure 7:**
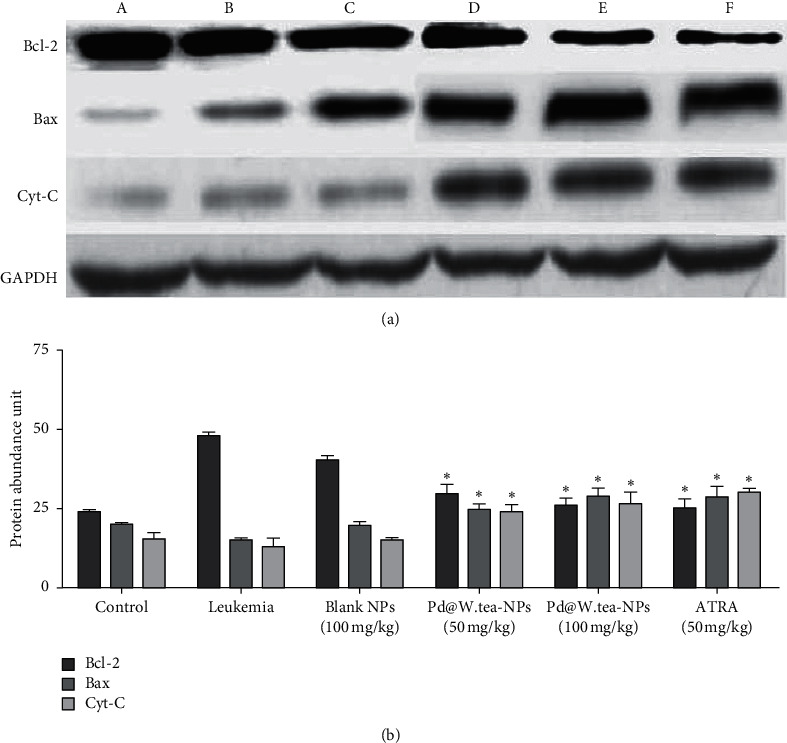
Expression of Bcl-2, Bax, cytochrome-C, and GAPDH (loading control) detected by western blotting in spleen from healthy, control BALB/c mice (A); mice with induced leukemia (B); mice with induced leukemia treated with 100 mg/kg blank Pd-NPs (C); mice with induced leukemia treated with 50 mg/kg Pd@W.tea-NPs (D); mice with induced leukemia treated with 100 mg/kg Pd@W.tea-NPs (E); and mice with induced leukemia treated with 5 mg/kg ATRA (F). The data shown are representative of multiple blots. Abbreviations: Cyt-C, cytochrome-C; GAPHD, glyceraldehyde 3-phosphate dehydrogenase. Protein abundance was measured by densitometry using ImageJ 148-JDK 6 software for *n* = 3 mice per group, each measured in triplicate. ^*∗*^*P* < 0.05 and ^*∗∗*^*P* < 0.05 by one-way ANOVA followed by Tukey's B-test. Values are mean ± SD relative to GAPDH (loading control).

Protein determination by western blot (Figure 7) and mRNA by RT-qPCR (Table 4) has revealed a significant increase in Bcl-2 and cleaved cytochrome-C and a significant reduction in Bax (consistent with stimulation of the caspase-induced pathway of apoptosis) in spleen from the groups treated with Pd@W.tea-NPs at both doses tested, as well as in the group treated with ATRA (positive control), compared with the untreated, leukemic group. In contrast, the group treated with blank NPs showed no differences from the untreated leukemic group.

## 4. Discussion

Over recent years, a plethora of plant part crude extracts and active compounds have been studied for their potential to generate inorganic NPs, and among them, *Camellia sinensis* (white tea) is the significant one [22]. Extract from the unfermented young tea leaves or unopened buds acts as an efficient reducing and capping agent to generate palladium nanoparticles in simple one-step green process [6]. The palladium nanoparticles generated (Pd@W.tea-NPs) were spherical, 6–8 nm in size and contained flavonoids and other phenols derived from the white tea extract. These nanoparticles were shown to have 1-diphenyl-2-picrylhydrazyl (DPPH), OH, and NO-scavenging properties *in vivo* using the MOLT-4 human leukemic cell line.

The current study aimed to explore if Pd@W.tea-NPs were effective against leukemia *in vivo*, using a mouse model of induced leukemia. Effects of Pd@W.tea-NPs that are consistent with anticancer activity in the mouse WEHI-3B leukemic cell line that we report complement our *in vivo* observations. Specifically, we found that both IC_50_ values and maximum reduction in cell viability for the action of Pd@W.tea-NPs over 24–72 h on WEHI-3B cells were lower than the equivalent values for normal mouse fibroblasts. This demonstrates a selectivity towards leukemic compared with normal cells, which is required for therapeutic efficacy. Notably, the IC_50_ values we measured (16.77, 10.25, and 7.55 *μ*g/ml for exposure over 24, 24, and 72 h, respectively) are similar to the values measured in human MOLT-4 leukemic cells (4.97, 6.25, and 7.14 *μ*g/ml, respectively, for exposure over the same time periods) [6]. This parity adds some confidence to the assertion that the positive anticancer effects we observed in the mouse model will translate to treatment of the disease in humans. We expected, and confirmed, that white tea extract-derived compounds incorporated into Pd@W.tea-NPs during their synthesis would confer cytotoxic effects towards cancer cells that are enhanced compared with “blank” Pd nanoparticles, generated in the absence of white tea extract. We previously demonstrated that polyphenolic constituents of white tea are incorporated into the Pd@W.tea-NPs6 and proposed these to be the most likely candidates.

Defects in the normal cell cycle arrest response to DNA damage may lead to development of cancer [23, 24], and induction of cell cycle arrest is an important element of the anticancer action of many chemotherapeutic agents used clinically. Control over the cell cycle has been proven as the main event in the process of cell division. The cell cycle regulators and check points such as G2/M checkpoint specifically is a target for anticancer therapy. Preventing entry into mitosis (M-phase) stimulates the induction of apoptotic pathways [24]. Our finding that Pd@W.tea-NPs, but not blank nanoparticles, induced G2/M phase arrest suggests that this action underlies, or at least contributes to, the measured effects on WEHI-3B cell viability.

Macroscopic and cellular features of the spleen observed in the mouse model of induced leukemia we used confirmed the presence of characteristic features of the disease that were ameliorated by Pd@W.tea-NPs administered orally at 2 different doses as well as by the anticancer drug ATRA, which is a common, effective therapeutic agent used to treat the disease in human patients. An increase in the size and weight of the spleen, most likely caused by the infiltration of leukemic cells [25–27], is one such feature of the disease. The observed accumulation of cells with abnormal morphology, characteristic of neoplastic cells, we observed in the diseased mouse spleen, and the increased abundance of cells positive for both the T-cell marker CD317 and the B-cell marker CD1918, which are potential indicators for disease severity [25], all point towards there being infiltration of leukemic cells, hence replicating this feature of the human disease. This validation of the experimental model adds strength to the argument that Pd@W.tea-NPs offer early promise as a potential treatment for human leukemia.

Blank Pd nanoparticles were not efficacious against these features of the disease demonstrating that, consistent with the effects measured *in vitro*, the content of the nanoparticles derived from the white tea extract provided efficacy. We did not test white tea extract alone in the animal model since the cytotoxic effects against WEHI-3B cells were far smaller than the effects of Pd@W.tea-NPs over the same concentration range and on a par with the effects of the blank nanoparticles. Thus, in line with the principles of 3R, it was considered better experimental design to exclude this test.

Since we observed effects of Pd@W.tea-NPs on the cell cycle in WHEI-3B cells that can lead to the induction of apoptosis, we posited that induction of apoptosis of leukemic cells in the spleen contributed to the effect of Pd@W.tea-NPs to reduce the size of the diseased spleen [28]. In apoptosis, Bcl-2 and Bax proteins activates the cascade of reactions by releasing cytochrome-C from the mitochondria which in turn forms apoptosome that helps in consecutive activation of caspases and eventually leads to cell death [29]. Particularly, in leukemia, Bcl-2/Bax ratio is an important determinant of cell survival [30]. These cytoplasmic proteins are also involved in retiring proliferating cells back to G0 phase of the cell cycle [31]. The increased Bcl-2/Bax ratio we observed in the spleen of the groups treated with Pd@W.tea-NPs and the increased level of cleaved cytochrome-C, indicating activation of the mitochondrial, caspase-induced pathway of apoptosis, is consistent with this assertion and replicated effects of ATRA we observed. In contrast, and commensurate with our other negative observations, blank nanoparticles did not induce these changes. The fact that Pd@W.tea-NPs replicated actions of ATRA, a current standard and effective treatment for leukemia, adds further weight to the argument that these NPs merit further investigation as future agents for leukemia treatment.

Alongside progression of work *in vivo* towards conducting clinical trials in patients, it would be worthwhile conducting further research *in vitro* to gain more knowledge of the delivery pathway of NP-delivered white tree extract components into the intracellular milieu. Specifically, resolving the question of whether NPs enter the cell and deliver their therapeutic cargo in soluble form to the cytosol, if active components are released at the cell membrane before entry into cells or if intracellular NPs per se are bioactive could guide formula optimisation.

## 5. Conclusions

In the current research, we have demonstrated *in vitro* and *in vivo* data on actions of Pd@W.tea-NPs that are consistent and that reveal actions likely to afford therapeutic efficacy against leukemia and likely to pivot on activation of the mitochondrial, caspase-induced pathway of apoptotic signaling. In all tests, Pd-NPs prepared in the absence of white tea extract lacked these actions, which demonstrates that components of the extract incorporated into the nanoparticles, likely to be polyphenolic compounds, contribute these actions. Thus, Pd@W.tea-NPs are promising candidates for leukemia therapy that merit further study in this specific context and also as possible agents for the treatment of other forms of cancer.

## Figures and Tables

**Table 1:** Serum liver and renal function parameters of healthy (negative) control mice or with induced leukemia after 28 days of treatment as stated.

Animal group	ALP (U/L)	ALT (U/L)	AST (U/L)	Urea (mmol/L)	Creatinine (*μ*mol/L)
Negative control	110.3 ± 2.1	60.9 ± 1.2	142.1.2 ± 1.3	7.85 ± 0.6	31.65 ± 0.8
Induced leukemia control	200.1 ± 1.1^*∗*^	155.55 ± 1.5^*∗*^	205.0.6 ± 0.9^*∗*^	12.55 ± 0.5^*∗*^	50.7 ± 2.3^*∗*^
Blank-NPs	195.01 ± 1.4^*∗*^	149.2 ± 1.1^*∗*^	197.5 ± 1.6^*∗*^	11.95 ± 0.55^*∗*^	51.6 ± 1.3^*∗*^
50 mg/kg Pd@W.tea-NPs	151.0 ± 0.6^Ψ^	100.3 ± 1.7^Ψ^	175.1.8 ± 0.8^Ψ^	9.05 ± 0.65^Ψ^	39.4 ± 0.4^Ψ^
100 mg/kg Pd@W.tea-NPs	128.4 ± 0.11^Ψ^	75.95 ± 1.45^Ψ^	155.1 ± 2.2^Ψ^	8.6.45 ± 0.25^Ψ^	35.23 ± 0.7^Ψ^
5 mg/kg ATRA	112.3 ± 1.5^Ψ^	62.0 ± 1.0^Ψ^	146.2 ± 0.35^Ψ^	8.1 ± 0.5^Ψ^	32 ± 0.9^Ψ^

^*∗*^ *P* < 0.05 compared with negative control, ^Ψ^*P* < 0.05 compared with induced leukemia control by one-way ANOVA followed by Tukey's B-test. ALP: alkaline phosphatase; ALT: alanine aminotransferase; AST: aspartate aminotransferase. Values are mean ± SD (*n* = 6).

**Table 2:** Spleen length (mm) and spleen weight (mg) in healthy (negative) control mice or with induced leukemia after 28 days of treatment as stated.

	Negative control	Induced leukemia control	100 mg/kg Pd@W.tea-NPs	50 mg/kg Pd@W.tea-NPs	Blank NPs	5 mg/kg ATRA
Length	220 ± 0.11	800 ± 0.5	325 ± 0.03^*∗*^	380 ± 0.22^*∗*^	733 ± 0.08	265 ± 0.67^*∗*^
Weight	17.5 ± 1.5	23.7 ± 1.4^*∗*^	19.4 ± 0.9^*∗∗*^	19.8 ± 1.0^*∗∗*^	22.1 ± 1.7	17.7 ± 1.1^*∗∗*^
	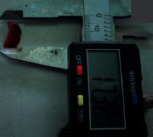	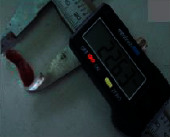	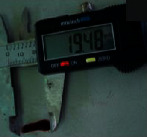	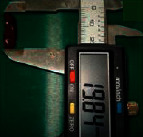	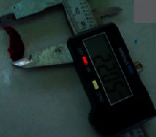	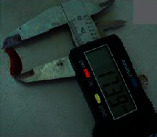

^*∗*^ *P* < 0.05 and ^*∗∗*^*P* < 0.005 compared to induced leukemia control by one-way ANOVA followed by Tukey's B-test. Representative photographs are shown below the relevant column. Values are mean ± SD of 6 freshly excised spleens.

**Table 3:** Histopathological scoring of spleen in mice with induced leukemia, or in healthy (negative) control animals, after 28 days of treatment as stated.

Animal group	Leukemic cells %	Nonleukemic cells %	Score	Indication
Negative control	0.0 ± 0.0	100.0 ± 0.0	0	Normal
Induced leukemia control	85.51 ± 1.4a	14.49 ± 0.7	4	Severe change
50 mg/kg Pd@W.tea-NPs	19.65 ± 0.45b	80.35 ± 0.55	1	Mild change
100 mg/kg Pd@W.tea-NPs	18.2 ± 0.25b	81.8 ± 0.15	1	Mild change
Blank-NPs	72.25 ± 2.1a	27.75 ± 1.5	4	Severe change
5 mg/kg ATRA	13.50 ± 0.16b	86.50 ± 0.26	1	Mild change

Values are mean ± SD, *n* = 4 sections. a - *P* < 0.05 compared with negative control; b–*P* < 0.05 compared with induced leukemia control by one-way ANOVA followed by Tukey's B-test. Sections were stained with H&E.

**Table 4:** Bcl-2, Bax, and cytochrome-C mRNA measured and normalized by GAPDH gene by RT-qPCR in spleen from healthy (negative) control mice or mice with induced leukemia after 28 days of treatment as stated.

mRNA	Control	Leukemia	Blank NPs (100 mg/kg)	Pd@W.tea-NPs (50 mg/kg)	Pd@W.tea-NPs (100 mg/kg)	ATRA (5 mg/kg)
Bcl-2	12.5 ± 0.11	33.01 ± 1.15	31.23 ± 0.75	25.25 ± 0.25^*∗*^	21.12 ± 0.44^*∗*^	16.98 ± 1.1^*∗*^
Bax	14.07 ± 1.7	11.34 ± 1.0	11.55 ± 0.35	25.24 ± 0.20^*∗∗*^	31.10 ± 0.17^*∗∗*^	42.10 ± 2.2^*∗∗*^
Cyt-C	12.0 ± 1.15	8.60 ± 0.75	9.25 ± 0.25	21.54 ± 0.30^*∗∗*^	26.45 ± 0.05^*∗∗*^	34.05 ± 2.1^*∗∗*^

Values are mean ± SD relative to GAPDH (loading control) measured by densitometry using ImageJ 148-JDK 6 software for *n* = 3 mice per group, each measured in triplicate. ^*∗*^*P* < 0.05 and ^*∗∗*^*P* < 0.05 by one-way ANOVA followed by Tukey's B-test.

## Data Availability

The data used to support the findings of this study are included within the article.
